# Effectiveness of Proximal Femur Nail in the Management of Unstable Per-Trochanteric Fractures: A Retrospective Cohort Study

**DOI:** 10.7759/cureus.58078

**Published:** 2024-04-11

**Authors:** Rizwan H Rashid, Marij Zahid, Fizzah Mariam, Moiz Ali, Hassan Moiz, Yasir Mohib

**Affiliations:** 1 Orthopedic Surgery, Aga Khan University Hospital, Karachi, PAK; 2 Orthopedics and Traumatology, Aga Khan University Hospital, Karachi, PAK

**Keywords:** outcome scores, proximal femur nailing, unstable peri-trochanter fractures, elderly trauma, proximal femur fractures

## Abstract

Background

Intertrochanteric (IT) fractures in the elderly demand surgical intervention for optimal recovery. While dynamic hip screw (DHS) is standard for stable fractures, its use in unstable cases is debated. Proximal femur nail (PFN) addresses unstable per-trochanteric fractures, boasting biomechanical advantages. Many studies favor PFN over DHS, despite concerns like screw migration. In resource-constrained developing nations, the choice of implant is pivotal. This research assesses proximal femur nailing outcomes for unstable fractures, providing insights for regional orthopedic protocols and contributing to tailored treatment guidelines in contexts with limited resources.

Objective

To assess the clinical and radiological outcomes in patients undergoing proximal femur nailing for unstable per-trochanteric fractures.

Material and Methods

This retrospective single-arm cohort study was conducted from January 2020 to July 2022. All the consecutive patients who underwent PFN for unstable per-trochanteric fractures were included in this study. Harris Hip Score (HHS) and ambulation status were recorded to evaluate functional outcomes. In contrast, the radiological outcome was assessed by calculating Radiographic Union Score for Hip (RUSH) scores at six weeks, three months, and six months post-operatively.

Results

A total of 48 patients were included in this study with equal gender distribution and a mean age of 66 years. The functional outcome was recorded with 2.1% (1), 33.3% (16), and 50% (24) of patients achieving full weight bearing (FWB) without pain at six weeks, three months, and six months respectively while 14.6% (7) of the patients never achieved FWB. The radiological outcome was assessed by calculating RUSH score with 6.3% (3), 43.8% (21), and 50% (24) of the patients achieving complete union at the end of six weeks, three months, and six months respectively. One patient (2.1%) experienced malunion.

Conclusion

PFN remains an optimal treatment modality for the fixation of unstable per-trochanteric fractures yielding promising functional and radiological outcomes.

## Introduction

Intertrochanteric (IT) fractures are extremely common in the elderly population and with increasing age expectancy, their incidence is increasing worldwide [1-3]. These fractures contribute to a significant burden on healthcare and are associated with high morbidity and mortality rates if left untreated [4]. Surgical fixation is standard management for these patients to achieve acceptable reduction and allow early mobilization, thereby decreasing the incidence of complications resulting from immobility [5].

There are various treatment options available for the fixation of IT fractures. Dynamic hip screw (DHS) is considered to be the standard of care when dealing with stable IT fractures [Arbeitsgemeinschaft für Osteosynthesefragen (AO) type 31-A1], however, its use in unstable fractures (AO types 31-A2 and A3) is still controversial [6, 7]. For fixation of unstable fractures, intra-medullary nailing devices including gamma nail and proximal femur nail (PFN) have been preferred. [2, 3, 7].

The proximal femur nail was designed by AO in 1996 for the treatment of unstable per-trochanteric fractures [8]. Since then, its use has been progressively increasing across the globe, owing to the decreased incidence of varus collapse and superior biomechanics compared to extramedullary devices [9]. Various studies have shown proximal femur nail antirotation (PFNA) to be superior to DHS in terms of better recovery and functional outcomes [10]. A meta-analysis has shown that PFNA involves less blood loss and fewer complications as compared to DHS [11]. However, studies have also reported complications such as migration of head screws, screw cut-out, and peri-implant fracture with the use of PFN [3, 12].

Consequently, there is an ongoing discussion about the most suitable implant for addressing unstable per-trochanteric fractures. This disagreement is particularly significant in developing nations, where limited financial and resource constraints make it challenging to keep up with the latest technological advancements. Therefore, the primary aim of this research was to evaluate the clinical and radiological results following the use of proximal femur nailing for treating unstable per-trochanteric fractures in our region.

## Materials and methods

This is a retrospective single-arm cohort study conducted at a tertiary care center in Pakistan aimed to investigate the outcomes of patients who underwent proximal femoral nail (PFN) insertion for unstable pertrochanteric fractures between January 2019 and July 2022. Inclusion criteria for the study comprised adults aged 18 and above diagnosed with unstable intertrochanteric (IT) fractures treated with PFN. Exclusion criteria were set to ensure the homogeneity of the study population and mitigate potential confounding factors. Patients with known coagulation disorders, a history of previous surgery for the same fracture, pathological fractures, and incomplete medical records were excluded from the analysis. The study protocol received approval from the Ethical Review Committee of the institution, ensuring adherence to ethical principles and guidelines governing research involving human subjects.

Data collection was conducted through a meticulous review of medical records for all eligible patients. Baseline demographic information, including age, gender, and comorbidities, was documented, along with intraoperative details such as surgical approach, implant type, and duration of surgery. Immediate complications, if any, were noted from the initial hospital visit, providing insights into the perioperative safety profile of PFN insertion. Subsequent follow-up clinic visits at six-week, three-month, and six-month intervals allowed for the assessment of ambulation status, a crucial indicator of functional recovery and rehabilitation progress post-surgery.

Functional outcomes were evaluated using the Harris Hip Score (HHS), a validated tool for assessing hip function and pain. Additionally, radiographic union, a key indicator of fracture healing, was assessed using the Radiographic Union Score for Hip (RUSH) at the six-month postoperative mark. A RUSH score cutoff of >18 was utilized as evidence of fracture union, providing quantitative data on the success of the surgical intervention in promoting bone healing. Complications were systematically categorized as early (occurring during the hospital stay) or late (occurring after discharge) and were meticulously documented from the medical records to capture the full spectrum of postoperative adverse events.

Statistical analysis was performed using Stata version 17 (Stata Statistical Software: Release 17. College Station, TX: StataCorp LLC.), employing appropriate tests to analyze categorical and quantitative variables. Categorical variables were expressed as frequencies and percentages, while quantitative variables were presented as mean ± standard deviation. The choice of statistical tests, including the Chi-square test or Fisher exact test for categorical variables and the Student’s t-test or Wilcoxon Rank sum test for continuous variables, was guided by the distributional properties of the data. Linear regression analysis was conducted to measure the association between the predictor variables of interest and outcome variables i.e. HHS and RUSH scores respectively. A significance level of p < 0.05 was applied to determine statistical significance, ensuring robustness and reliability in the interpretation of study findings.

Overall, the study design and analysis approach was meticulously crafted to provide comprehensive insights into the outcomes and complications associated with PFN insertion for unstable pertrochanteric fractures. By elucidating the effectiveness and safety profile of this surgical intervention in a real-world clinical setting, the study aims to inform evidence-based decision-making and optimize patient care strategies in the management of pertrochanteric fractures, ultimately contributing to improved clinical outcomes and patient satisfaction.

## Results

A total of 48 patients were included in this study with equal gender distribution and a mean age of 71 years [interquartile range (IQR) = 56-80]. The majority of the patients had some underlying co-morbid conditions (e.g. diabetes mellitus, hypertension, etc.) and had sustained a low-velocity trauma (ground-level fall). Fractures classified using Evans classification were either Group 3 (56.3% - 27 patients) or Group 4 (43.8% - 21 patients). With regards to operative parameters, the mean duration of surgery was noted to be 59.7 ± 16 minutes, whereas the mean blood loss was 136.2 ± 83.2ml. A significant drop in hemoglobin level was noted post-operatively as compared to pre-operative values (p=<0.001). Details of baseline characteristics are depicted in Table 1.

**Table 1:** Baseline characteristics of study population (n=48) *ASA = American Society for Anesthesiologist, **PCV = Packed Cell Volume.

Characteristics	Frequency (%)
Gender	
Male	24 (50)
Female	24 (50)
Co-morbidities	
Yes	37 (77.1)
No	11 (22.9)
ASA* Status	
I	7 (14.6)
II	20 (41.7)
III	21 (43.8)
Mechanism of Injury	
High Energy	12 (25)
Low Energy	36 (75)
Fracture Classification (Evans)	
Group 3	27 (56.3)
Group 4	21 (43.8)
Anesthesia	
General	47 (97.9)
Spinal	1 (2.1)
Post-operative PCV** Transfusion (units)	
0	25 (52.1)
1	8 (37.5)
2	5 (10.4)

**Figure 1:**
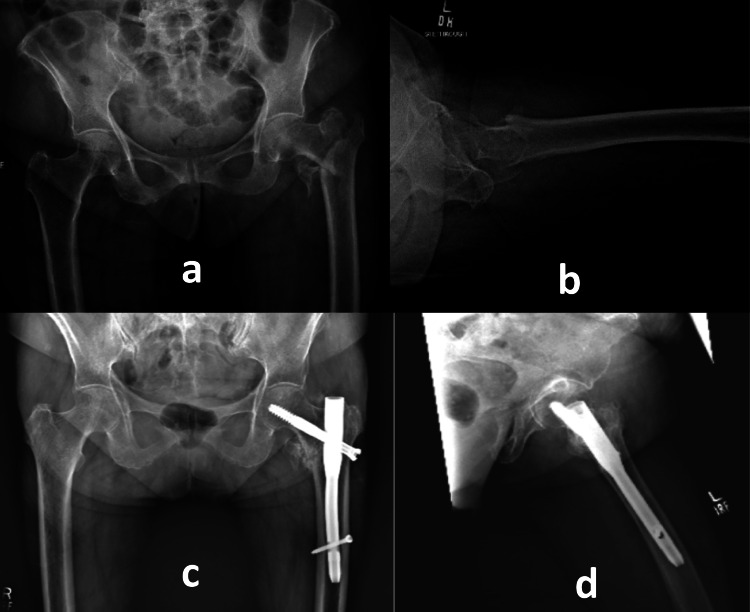
Pre-operative radiographs (AP view and lateral view) of a 75-year-old patient showing unstable per-trochanteric fracture of left femur (a & b) and Post-operative radiographs (AP view and lateral view) at 6 months showing Intertrochanteric fracture of left femur fixed via CRIF with Intramedullary nail showing radiological signs of healing with RUSH score of 20 and HHS of 80 (c&d).

During the early post-operative period, acute kidney injury (AKI), acute gastroenteritis (AGE), and development of hospital-acquired pneumonia (HAP) was observed in two patients (4.2%). All of these complications were managed with appropriate treatment involving the concerned subspecialties. The majority of the patients ambulated full weight bearing without pain by the six-month follow-up, however, there were seven patients (14.6%) who still had complaints of pain on full weight bearing. Six out of these seven patients were able to tolerate partial weight bearing whereas one patient was still ambulating without any weight bearing on the injured side at the final follow-up. Figure 1 illustrates radiographs of one of the cases.

The mean HHS at the six-month follow-up was 67.1 ± 13.4 while the mean RUSH score was 23.9 ± 3.67. Forty-five out of the 48 patients were observed to have a RUSH score >18, indicating an overall union rate of 93.8%. Out of the three patients who did not achieve complete union, one experienced malunion at three months and two patients had screw cut out (at six weeks and three months respectively). No significant association was found between demographic characteristics and either the HHS or RUSH scores as shown in Table 2.

**Table 2:** Association between demographic characteristics and HHS and RUSH score HHS = Harris Hip Score, RUSH = Radiographic Union Score for Hip, ASA = American Society for Anesthesiologists, Independent Sample T-test and Mann-Whitney U-test were used to evaluate associations

Characteristics	Significance (p-value)	
	HHS	RUSH
Gender	0.098	0.14
Co-morbidities	0.62	0.76
ASA Status	0.60	0.63
Mechanism of Injury	0.95	0.85
Fracture Classification	0.96	0.48

No significant correlation was noted between age and HHS (-0.076) and RUSH score (-0.054), with p-values of 0.72 and 0.61 respectively. Similarly, no significant association was observed when baseline characteristics were compared with the presence or absence of functional or radiological union as represented in Tables 3, 4 respectively. Linear regression analysis was conducted to measure the association between the predictor variables of interest and outcome variables i.e HHS and RUSH scores respectively which did not show any significant variability. HHS and RUSH scores were transformed into binary variables and logistic regression analysis was conducted but failed to conclude any significant final model. This in turn might be due to a small sample size and multicolinearity.

**Table 3:** Association of demographic characteristics with functional healing *ASA = American Society for Anesthesiologists, Chi-square test was used to evaluate associations.

Characteristics	Functional Healing (%)		Significance (p-value)
	Yes	No	
Gender			0.21
Male	22 (91.7)	2 (8.3)	
Female	19 (79.2)	5 (20.8)	
Co-morbids			0.49
Yes	31 (83.8)	6 (16.2)	
No	10 (90.9)	1 (9.1)	
ASA* Status			0.43
I	7 (100)	0 4 (20.0)	
II	16 (80.0)	3 (14.3)	
III	18 (85.7)		
Mechanism of Injury			0.43
High Energy	11 (91.7)	1 (8.3)	
Low Energy	30 (83.3)	6 (16.7)	
Fracture Classification			0.36
Type 3	24 (88.9)	3 (11.1)	
Type 4	17 (81.0)	4 (19.0)	

**Table 4:** Association of demographic characteristics with Radiological Union *ASA = American Society for Anesthesiologists, Chi-square test was used to evaluate associations.

Characteristics	Radiological Union (%)		Significance (p-value)
	Yes	No	
Gender			0.11
Male	24 (100)	0	
Female	21 (87.5)	3 (12.5)	
Co-morbids			0.45
Yes	34 (91.9)	3 (8.1)	
No	11 (100)	0	
ASA* Status			0.64
I	7 (100)	0	
II	19 (95.0)	1 (5.0)	
III	19 (90.5)	2 (9.5)	
Mechanism of Injury			0.41
High energy	12 (100)	0	
Low energy	33 (91.7)	3 (8.3)	
Fracture Classification			0.41
Type 3	26 (96.3)	1 (3.7)	
Type 4	19 (90.5)	2 (9.5)	

## Discussion

The management of unstable per-trochanteric fractures has always posed a significant challenge to orthopedic surgeons; however, it has evolved significantly during the past few decades. Fixation with dynamic hip screw (DHS) was regarded as the gold standard treatment paradigm but proximal femur nailing has now gained immense popularity around the globe as it proves to be superior biomechanically attributing to the fact that it reduces the distance between the implant and the joint [13]. Previous literature concludes that intramedullary nailing via PFN in comparison with extramedullary fixation via DHS offers lesser operative time, decreased blood loss, early mobility, and a shorter hospital stay thereby resulting in lesser postoperative complications and decreased morbidity [10,11,13].

According to the findings of our study, at the end of six months, the majority of the patients achieved a mean HHS of 67, thereby concluding that PFN renders an excellent functional outcome in unstable per-trochanteric fractures, supporting the previous literature. Gupta et al. reported that 66.2% of the patients achieved excellent results. [14]. Similarly, Haq et al. in their study concluded that cephalomedullary fixation via PFN nail, in addition to being a minimally invasive technique, has also proven to yield satisfactory functional outcomes with 66% of their study subjects yielding a mean HHS of 81.53 points at one year [15]. In a previous study (India), the functional outcomes of various surgical modalities for the treatment of unstable intertrochanteric fractures were assessed using HHS, and the patients who had undergone PFN fixation had a mean score of 88.25 points with a p-value of 0.015 proving that this technique yields significantly better results when compared with other modalities [16]. Mehta et al. reported that 68.33% of the patients in their study reported excellent to good outcomes [17]. The HHS reported in our study is lower in comparison with the existing literature because the majority of the patients were old age with poor baseline mobility.

PFN has been associated with considerably reduced surgical duration and blood loss compared to extramedullary fixation [6]. A meta-analysis conducted in 2014 showed the mean surgical duration to range from 51 to 64 minutes for PFN and from 65 to 99.6 minutes for DHS, with the mean duration of PFN being 21 minutes less than DHS and the difference being statistically significant (p = 0.003) [6]. With regards to blood loss, studies have shown per-operative blood loss to range from 123 to 320 ml for PFN compared to 269 to 480 ml for DHS, with the difference being statistically significant (p = 0.0001) [6]. The mean surgical duration of 59.7 minutes and blood loss of 136.2 ml noted in the current study is also comparable to internationally reported literature [18-20]. This demonstrates that PFN is not only better in terms of clinical outcome but it is also effective based on multiple intraoperative parameters.

Proximal Femur Nailing remains to be an optimal treatment modality for unstable per-trochanteric fractures with a better complication profile when compared with other modalities. Huang et al. concluded that there was a significant difference in the incidence of postoperative complications between PFNA, DHS, and proximal femur locking compression plate (PFLCP) [21]. According to the findings of our study, two patients experienced screw cuts out and one patient experienced malunion. The previous literature has reported the complication rates of PFN up to 31% with screw cut-out being one of the significant postoperative complications [2, 22]. In a study by Schipper et al., screw cut out remained the most frequent complication with a rate of 7% owing to the knife effect of PFN [23].

The results of our study are consistent with the previous literature and have shown that PFN remains a reliable option for the treatment of unstable per-trochanteric fractures rendering a satisfactory clinical and radiological outcome, although optimum fracture reduction and the correct placement of the implant is essential.

Limitations of our study include its retrospective nature and consecutive sampling strategy, which can lead to information and selection bias. This is also a single center-based study with a relatively smaller sample size, possibly limiting the statistical power of the study to detect weaker effects. Additionally, only one treatment modality for unstable per-trochanteric fractures was studied, resulting in a lack of a control group to directly compare outcomes. A study with a larger patient population and a comparison of PFN with alternative techniques to treat intertrochanteric fractures, such as DHS, would strengthen the cause of the study.

## Conclusions

Our study emphasizes the effectiveness of the proximal femur nail (PFN) in managing unstable per-trochanteric fractures at our tertiary center. The use of PFN exhibited efficient intra-operative parameters with a mean surgical duration of 59.7 minutes and a mean blood loss of only 136 ml, aligning well with international standards. The overall complication rate of 18.8% was comparable to global literature, indicating PFN's reasonable complication profile. Satisfactory functional outcomes were observed, with a mean HHS of 67.1 at the six-month follow-up, delineating PFN's ability to restore mobility and functionality. Radiological union, indicated by a RUSH score >18 in 93.8% of patients, further supports PFN's efficacy in promoting fracture healing. These findings reaffirm PFN as a reliable and effective treatment option, even in resource-limited healthcare settings, and contribute valuable insights to the fracture management debate. However, multicentric studies with a larger sample size and comparative analysis are essential to provide a more comprehensive understanding of PFN outcomes to facilitate evidence-based treatment strategies for unstable per-trochanteric fractures.
